# IQGAP1 Mediates Hcp1-Promoted *Escherichia coli* Meningitis by Stimulating the MAPK Pathway

**DOI:** 10.3389/fcimb.2017.00132

**Published:** 2017-04-19

**Authors:** Mingna Zhao, Lingfei Zhang, Shaogang Lv, Chenzi Zhang, Lin Wang, Hong Chen, Yan Zhou, Jiatao Lou

**Affiliations:** ^1^Department of Laboratory Medicine, Shanghai Chest Hospital, Shanghai Jiao Tong UniversityShanghai, China; ^2^Center for RNA Research, State Key Laboratory of Molecular Biology–University of Chinese Academy of Sciences, Institute of Biochemistry and Cell Biology Shanghai Institutes for Biological Sciences Chinese Academy of SciencesShanghai, China; ^3^Department of Anatomy, Histology and Embryology, Shanghai Medical College, Fudan UniversityShanghai, China; ^4^Department of Microbiology and Immunobiology, Harvard Medical SchoolBoston, MA, USA

**Keywords:** IQGAP1, HCP1, *Escherichia coli*, meningitis, MAPK pathway

## Abstract

*Escherichia coli*-induced meningitis remains a life-threatening disease despite recent advances in the field of antibiotics-based therapeutics, necessitating continued research on its pathogenesis. The current study aims to elucidate the mechanism through which hemolysin-coregulated protein 1 (Hcp1) induces the apoptosis of human brain microvascular endothelial cells (HBMEC). Co-immunoprecipitation coupled with mass spectrometric (MS) characterization led to the identification of IQ motif containing GTPase activating protein 1 (IQGAP1) as a downstream target of Hcp1. IQGAP1 was found to be up-regulated by Hcp1 treatment and mediate the stimulation of HBMEC apoptosis. It was shown that Hcp1 could compete against Smurf1 for binding to IQGAP1, thereby rescuing the latter from ubiquitin-dependent degradation. Subsequent study suggested that IQGAP1 could stimulate the MAPK signaling pathway by promoting the phosphorylation of ERK1/2, an effect that was blocked by U0126, an MAPK inhibitor. Furthermore, U0126 also demonstrated therapeutic potential against *E. coli* meningitis in a mouse model. Taken together, our results suggested the feasibility of targeting the MAPK pathway as a putative therapeutic strategy against bacterial meningitis.

## Introduction

Bacterial meningitis is a type of inflammation caused by an infectious bacterial species in the meninges, which consist of three layers of protective membranes that surround the brain and the spinal cord (Grimwood et al., 2000). Common causative agents of bacterial meningitis include *Streptococcus pneumoniae, Haemophilus influenzae, Streptococcus agalactiae, Escherichia coli*, etc (Schut et al., 2008; Brouwer et al., 2010). Despite the development of vaccines and antibiotics that have provided highly effective preventative and treatment solutions, bacterial meningitis remains a dangerous disease. For example, ~4,100 cases of bacterial meningitis were recorded in the United States between 2003 and 2007 with a mortality rate of 12.2% (Thigpen et al., 2011). Clinical and laboratory data have demonstrated that conventional antimicrobial therapeutics often lead to less-than-satisfactory outcome in bacterial meningitis patients (Kim, 1985; Flores et al., 2013), highlighting the gaps in the current understanding of the pathogenic mechanism of the disease.

There is mounting evidence that the type VI secretion system (T6SS) is implicated in the pathogenicity of many infectious bacterial agents (Bingle et al., 2008; Silverman et al., 2012). T6SS can mediate the injection of bacterial virulence factors into the recipient cells, leading to the latter's destruction. The core structure of T6SS comprises 13 essential subunits and could contain additional accessory components (Bingle et al., 2008; Cascales and Cambillau, 2012). Hemolysin-coregulated protein (Hcp) and valine glycine repeat (VgrG) are both considered to be the hallmarks of T6SS and show considerable homology to essential proteins that form the punctuating injector of a typical bacteriophage (Brunet et al., 2014). Since the discovery of its first member in *V. cholerae* as part of the T6SS machinery (McCracken et al., 1984; Ishikawa et al., 2009), the Hcp family proteins have been identified in a variety of bacterial species. An Hcp1/Hcp2 double knockout of *V. cholerae V52* was generated by transposon mutagenesis and showed no virulence on *Dictyostelium discoideum* (Pukatzki et al., 2006). Hcp was also detected in patients with pulmonary secretions of cystic fibrosis (CF) in possible association to the pathogenicity of the causative agent *Pseudomonas aeruginosa* (Mougous et al., 2006). Similarly, Hcp family proteins were abundantly secreted by *Burkholderia mallei* when the two-component transcriptional regulatory system VirAG, required for maximal virulence of the pathogen, was overexpressed (Schell et al., 2007). In another study, *Agrobacterium tumefaciens* lacking Hcp was 20–30% less efficient in promoting tumorigenesis (Wu et al., 2008).

Recently, we reported that the T6SS and Hcp family proteins were associated with the invasion of human brain microvascular endothelial cells (HBMEC) by meningitis-causing *E. coli* K1 RS218 (Zhou et al., 2012). This constituted the first experimental evidence for the involvement of Hcp proteins in the development of bacterial meningitis. The two Hcp proteins identified in *E. coli*, namely Hcp1 and Hcp2, were suggested by deletion experiments to play different roles. Hcp2 was believed to assist in the contact and invasion of HBMEC cells, whereas Hcp1 was likely to be directly responsible for the induction of their apoptosis. However, no conclusive evidence in support of these proposed molecular roles has been obtained; In this study, we aim to get further insight into the exact mechanism for the Hcp1-induced HBMEC cell apoptosis and have identified that IQGAP1-induced phosphorylation of extracellular signal-related kinases 1/2 (ERK1/2) was responsible for Hcp1-promoted cell apoptosis of HBMEC cells. We also evaluated the effect of U0126, an inhibitor of the mitogen-activated protein kinases (MAPK) signaling pathway, on alleviating the apoptosis-inducing effect of Hcp1 and IQGAP1.

## Materials and methods

### Cell culture, bacterial culture, and reagents

Human brain microvascular endothelial cell line (HBMEC) obtained from American Type Culture Collection was grown in RPMI 1640 medium supplemented with 10% heat-inactivated fetal bovine serum (Gibco, USA), 2 mM glutamine, 1 mM pyruvate, 100 g/mL penicillin, 100 g/mL streptomycin, 1% essential amino acids, and 1% MEM vitamin solution (Gibco, USA). The meningitis-causing *E. coli* strain RS218, whose genome contains a T6SS gene cluster, was chosen as the model bacterial strain. In addition, a hcp1-deleted mutant RS218 strain, named RS218 Δhcp1, was included as a negative control. Both RS218 and RS218 Hcp1 mutant were grown in LB broth with appropriate antibiotics. The deletion of hcp1 from RS218 was performed based on a previously described protocol (Datsenko and Wanner, 2000; Zhou et al., 2012).

### Small interfering RNA (siRNA) transfection and cell treatment

siRNA reagents and their cognate control RNAs were purchased from Ribobio, China. The sequences are as follows:

IQGAP1 siRNA: 5′-UUAUCGCCCAGAAACAUCUUGUUGG-3′;
ARHGAP24 siRNA: 5′-ACCGAGAGAGGAAACACAATA-3′;
smurf1 siRNA: 5′-GCAUCGAAGUGUCCAGAGAAG-3′;
scrambled siRNA: 5′-UUCUCCGAACGUGUCACGU-3′.

Transfection was performed using Lipofectamine 2000 (Thermo, USA) according to the manufacturer's instructions. For IQGAP1 rescue experiment, a Pcmv-3XFLAG vector harboring IQGAP1 gene coding region was co-transfected with the IQGAP1 siRNA. For Hcp1 and Hcp2 treatment, HBMEC cells were incubated with 50 μg/mL of Hcp1 or Hcp2 for 48 h at 37°C in a 5% CO_2_ incubator. For infection with wild type or the mutant strain of RS218, HBMEC cells were infected with 10^7^ cfu *E. coli* at 37°C for 90 min in the same culture medium as described above. For MG132 treatment, 50 mM of MG132 was added to the cell culture 6 h before the cells were harvested. For U0126 treatment, the cells were pre-treated with U0126 (20 μM) for 24 h before Hcp1 incubation.

### Mouse model of meningitis

C57BL/6 mice were purchased from SLACS (Shanghai, China). This study was carried out in accordance with the guidelines of the Institutional Animal Care and Use Committee of the Institute for Biochemistry and Cell Biology, Shanghai Institutes for Biological Sciences, China. The protocol was approved by the Institute of Biochemistry and Cell Biology, Shanghai Institutes for Biological Sciences, China. C57BL/6 mice (5-day-old) were inoculated intranasally with 10 μL of bacterium solution containing 10^5^ cfu *E. coli*. The uninfected and U0126 control group were injected with 10 μL of isotonic saline. Both treatment and U0126 control group were subjected to 20 μL intraperitoneal injections of U0126 (Cell Signaling Technology, USA) at the dose of 3 mg/kg each, delivered at the following time points: 24 h prior to the inoculation of *E. coli*, at the time of inoculation, as well as 24 and 48 h after inoculation. After 72 h following the inoculation, the mice were euthanized and the brains were harvested. One half of each brain specimen was fixed in 4% PFA for histopathological analysis. The remaining half was homogenized for cytokine assays (Zwijnenburg et al., 2007; Mittal et al., 2010).

### RNA isolation and quantitative reverse transcription PCR (qRT-PCR) assays

RNA isolation and qRT-PCR analysis were performed as previously described (Wang et al., 2014). Total RNA was extracted from cells with TRIzol reagent (Thermo Fisher Scientific, USA). The mRNA levels were determined by qRT-PCR using SYBR Green (Takara, Japan) with GAPDH as an internal standard. The qRT-PCR primer sequences are as follows:

IQGAP1 forward primer: 5′-GGGACCAACCAAAGTGTGTCAAC-3′;
IQGAP1 reverse primer: 5′-CTGCTCATTATTGCCTGTCTTGGA-3′;
ARHGAP24 forward primer: 5′-CTGGTTCTGGAAGTTCTCGG-3′;
ARHGAP24 reverse primer: 5′-CTTTGACTGTGGGGAGAAGC-3′;
GAPDH forward primer: 5′-TCATCATCTCTGCCCCCTCT-3′;
GAPDH forward primer: 5′-CTGGGTGGCAGTGATGGCAT-3′.

### Preparation of nuclear extracts

The nuclear fraction of HBMEC cells was extracted using NE-PER Nuclear and Cytoplasmic Extraction Reagents (Thermo Scientific, USA) according to the protocol provided by the manufacturer. Briefly, 10^6^ PBS-washed cells were transferred to a 1.5 mL tube and pelleted by centrifugation at 500 g for 2–3 min followed. A total volume of 200 μL ice-cold CER I was added to the cell pellet. The tube was then vortex vigorously and incubated on ice for 10 min, followed by the addition 11 μL ice-cold CER II. The tube was vortexed again and centrifuged for 5 min at 16,000 g. The supernatant was collected as the cytoplasmic extracts. Meanwhile, the insoluble fraction was re-suspended in 200 μL ice-cold NER, vortex vigorously, and centrifuged for 5 min at 12,000 g. The supernatant was collected as the nuclear extract.

### Immunoprecipitation

After being treated with Hcp1, HBMEC cells were homogenized in lysis buffer (50 mM Tris-HCl at pH 7.5, 1% NP-40, 150 mM NaCl, 5 mM EDTA, and 5 mM EGTA) containing protease inhibitor cocktail. After centrifugation at 16,000 g and 4°C for 10 min, cell lysates were incubated with antibody-coupled Protein A beads (Sigma, USA) according to the manufacturer's instructions. The resultant protein complexes were washed in lysis buffer and diluted in SDS loading buffer for standard Western blot or mass spectrometric analysis (Zhao et al., 2013).

### Mass spectrometry

The co-immunoprecipitated protein complexes that were pulled down by His antibody or IgG control were separated on a 5–20% gradient SDS-PAGE gel, which was subsequently stained with Silver Quest (Thermo Fisher Scientific, USA). The identified protein bands were excised from the gel and subjected to in-gel digestion, in which the dried gel pieces were rehydrated and digested overnight at 30°C in 25 mM NH_4_HCO_3_ solution containing 12.5 ng/mL trypsin (Promega, USA). Peptide sequence analysis was performed on an Agilent LC system coupled to a Finnigan LTQ Mass spectrometry (Thermo Fisher Scientific, USA) at Core Facility of Molecular Biology, Institute of Biochemistry and Cell Biology (SIBCB).

### Western blot

Western blotting was performed as previously described (Jiang et al., 2010; Wang et al., 2014). Anti-rabbit-IQGAP1 antibody was purchased from Proteintech, USA. Anti-rabbit-His, anti-rabbit-UAP56, anti-rabbit-ARHGAP24, anti-rabbit-Cleaved-Caspase-8, anti-rabbit-Cleaved-Caspase-3, anti-rabbit-Cleaved-Caspase-9, anti-rabbit-p-ERK1/2, anti-rabbit-ERK1/2, and anti-rabbit-smurf1 antibodies were purchased from Cell Signaling Technology, USA. Anti-β-actin antibody was purchased from Sigma-Aldrich, USA.

### Immunofluorescence assay and histological examination

Immunofluorescence assay was performed as previously described (Jiang et al., 2010; Wang et al., 2014). The slides of treated cells or frozen section of brain tissues were fixed in 4% paraformaldehyde and permeabilized with 0.5% Triton X-100 in PBS. Non-specific binding was blocked with 5% normal goat serum and 2% BSA in PBS. The blocked cells or tissues were then incubated separately with each of the primary antibodies of choice (Rabbit anti-IQGAP1, anti-His, anti-smurf1, and anti-Ly6G) over night at 4°C. After washing with PBS five times, the cells or tissues were incubated with the secondary antibody, FITC-conjugated goat anti-rabbit IgG (Jackson, USA), for 2 h at 4°C in darkness. After washing with PBS five times, the nuclei were stained with DAPI for 5 min at room temperature in darkness. The slides were observed under a fluorescence microscope (BX51, Olympus, Japan). Fluorescence densities were quantified using Image J software as previously described (Zhao et al., 2013). For histological examination, paraformaldehyde-fixed brain tissues sections were stained with hematoxylin and eosin (H&E) based on a previously described protocol (Zhang et al., 2015).

### Overproduction and purification of the Hcp proteins

Hcp1 and Hcp2 were purified following a previously described protocol (Parthasarathy et al., 2007; Zhou et al., 2012; Douzi et al., 2014). Briefly, wild-type *E. coli* RS218 cells were transformed with pQE80-hcp1 or pQE80-hcp2 vector. The transformants were cultivated in 15 mL of LB medium overnight and re-inoculated into 1.5 L of LB on the second day. The resultant culture was grown at 37°C to an OD600 of 0.5. The culture was then cooled to 25°C, induced with 100 μM isopropyl-β-D-thiogalactopyranoside (IPTG) and grown for an additional 4 h. Cells were harvested and resuspended in 20 mL buffer A containing 50 mM Tris-HCl at pH 8.0, 300 mM NaCl, and 0.1 mg/mL lysozyme, followed by a 30 min incubation on ice, before being lysed by sonication. Insoluble materials were removed by centrifugation at 14,000 rpm. Hcp proteins were purified via affinity chromatography by incubating with 5 mL of Ni-nitrilotriacetic acid agarose (QIAGEN, Valencia, CA) overnight at 4°C. The resultant slurry was loaded onto two disposable standing columns, each of which was washed with 12 mL buffer A and 12 mL buffer B containing 50 mM Tris-HCl at pH 8.0, 300 mM NaCl, and 5 mM imidazole. The target protein was eluted with buffer C containing 50 mM Tris-HCl at pH 8.0, 150 mM NaCl, and 300 mM imidazole, with a total volume of 1 mL eluent collected. The eluted protein was electrophoresed on a 10% sodium dodecyl sulfate–polyacrylamide gel electrophoresis (SDS-PAGE) gel, and fractions containing His6-FliC were pooled, dialyzed against buffer E (10 mM Tris-HCl [pH 8.0], 10 mM NaCl) using Slide-A-Lyzer dialysis cassettes (Pierce, Rockford, IL), and concentrated to 2 mg/ml with a Centricon YM-10 centrifugal concentrator (Millipore, Billerica, MA). The dialyzed protein (0.5 μg) was then electrophoresed on a 12% bis-Tris SDS-PAGE gel to evaluate its purity.

### TUNEL staining

TUNEL assay was performed by using the Fluorometric TUNEL System (Promega, USA) according to the protocol provided by the manufacturer. Briefly, 50 μL of rTdT Incubation Buffer was added to the fixed cells on slides. Then, the slides were incubated under humidified atmosphere for 60 min at 37°C in darkness and rinsed three times with 2 × SSC for 5 min each time. The slides were subsequently stained with DAPI and observed under a fluorescence microscope (BX51, Olympus, Japan). TUNEL-positive cells were indicated by the emission of green fluorescence and the nuclei were visualized by the blue fluorescence. Ratio of the number of TUNEL-positive neurons to the total number of nuclei was calculated.

### Brain cytokine assays

Murine brains were homogenized in 4 vol of sterile saline, followed by incubation in an equal volume of lysis buffer containing 300 mmol/L NaCl, 15 mmol/L Tris-HCl at pH 7.4, 2 mmol/L MgCl_2_, 2 mmol/L CaCl_2_, 2 mmol/L Triton X-100, 20 ng/mL pepstatin A, 20 ng/mL leupeptin, and 20 ng/mL aprotinine. After 30 min of incubation, the resultant brain homogenates were centrifuged for 15 min at 1,500 g. The supernatant was collected and stored at –20°C for brain cytokine assays. The levels of murine cytokines, including those of chemokine (C-X-C motif) ligand 1 (KC), macrophage inflammatory protein-2 (MIP-2), interleukin-1α (IL-1α), interleukin-1β (IL-1β), interleukin-6 (IL-6), and tumor necrosis factor-α (TNF-α), were measured using commercially available ELISAs Kit (R&D Systems, USA) according to the manufacturer's instructions (Parthasarathy et al., 2007; Zwijnenburg et al., 2007).

## Results

### Internalization of Hcp1 promotes apoptosis of HBMEC cells

**Figure 1:**
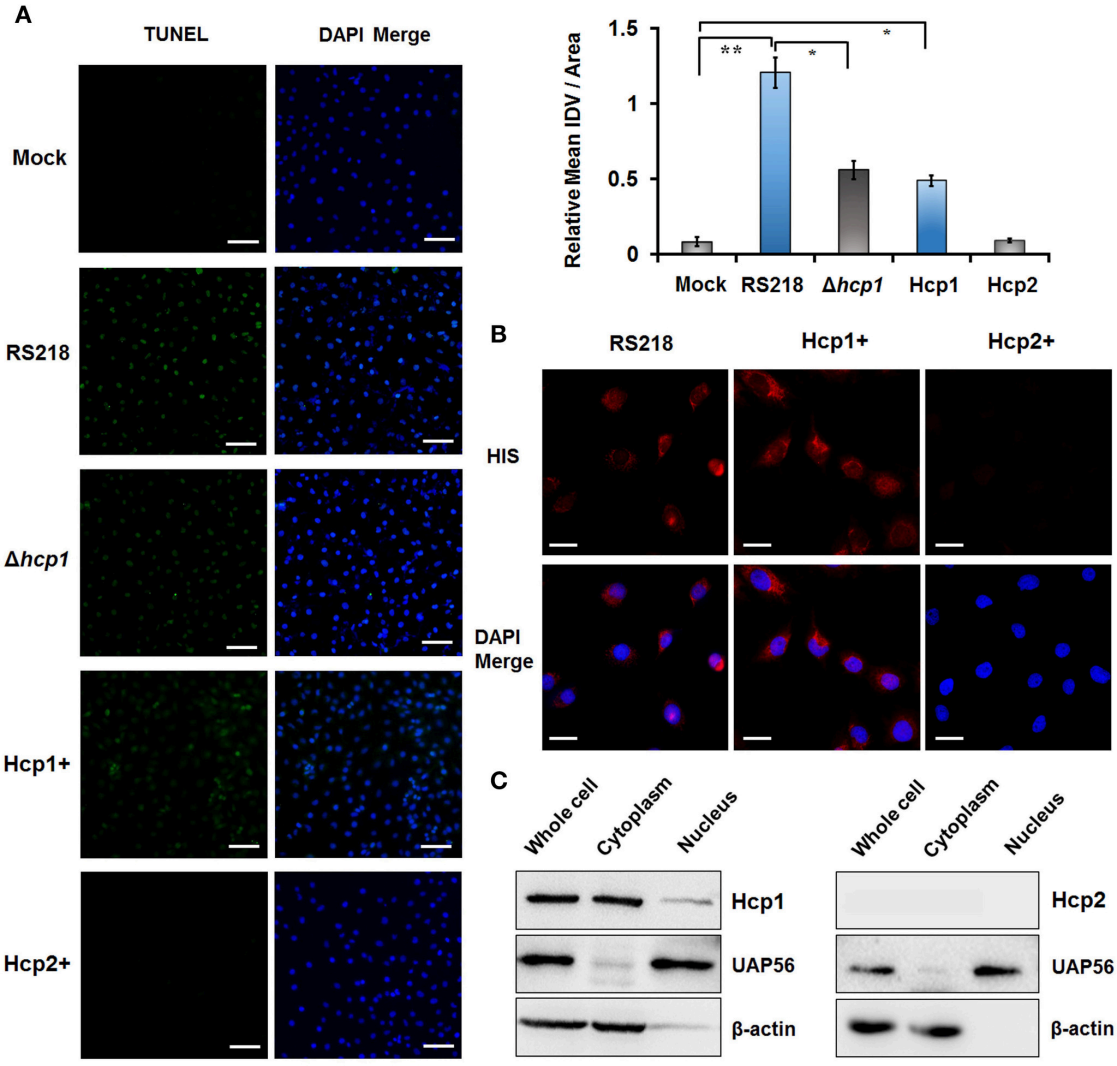
**Transport of Hcp1 into the cytoplasm of HBMEC cells leads to increased apoptosis. (A)** TUNEL assays were conducted on HBMEC cells treated with PBS solution (Mock), wild-type RS218 (RS218), Δhcp1 mutant of RS218 (Δhcp1), Hcp1 (Hcp1+), or Hcp2 (Hcp2+). Left panel: TUNEL-positive cells were indicated by the green fluorescence. DAPI was used as a counterstain to mark the nuclear regions of the cells. Right panel: Relative mean integrated density value (IDV) of TUNEL signal (green) from three independent experiments. Scale bar, 50 μm. ^*^P < 0.05, ^**^P < 0.01. **(B)** Fluorescence microscopy showing the internalization of Hcp1 by HBMEC cells. The presence of the Hcp proteins (left: Hcp1; right: Hcp2) was detected by rabbit anti-His antibody and indicated by the red fluorescence. Scale bar, 50 μm. **(C)** Localization of Hcp1 (top) and Hcp2 (bottom) in the treated HBMEC cells as determined by Western blotting. UAP56 and β-actin were used as nuclear and cytosolic marker, respectively.

We began our study by seeking to obtain further evidence that Hcp1, not Hcp2, is directly responsible for inducing apoptosis in HBMEC cells. To this end, HBMEC cells were incubated separately with His-tagged Hcp1 or Hcp2, followed by the detection of apoptosis via TUNEL assay. DAPI was used as a counterstain to mark cell nuclei. As illustrated in Figure 1A, the merged microscopic images clearly indicated that the incubation with Hcp1, but not Hcp2, triggered widespread cell apoptosis. As further evidence, the deletion of Hcp1 from *E. coli* RS218, a well-established meningitis-causing strain, resulted in significantly diminished apoptosis of HBMEC cells (Figure 1A). Importantly, we observed similar apoptotic levels between HBMEC cells infected by wild-type RS218 and those treated with Hcp1 (Figure 1A). In addition, immunofluorescence staining using rabbit anti-His primary antibody and FITC-conjugated goat anti-rabbit IgG secondary antibody also verified the presence of Hcp1 in the treated HBMEC cells (Figure 1B), whereas almost no Hcp2 was observed. Consistently, Western blot showed no visual evidence that Hcp2 was intracellularly accumulated (Figure 1C), whereas Hcp1 was found to be visibly present in the whole-cell extracts and primarily localize in the cytoplasm (Figure 1C). These results offered direct experimental proof that Hcp1, rather than Hcp2, enters the HBMEC cells and triggers the downstream apoptotic pathway.

### IQGAP1 is involved in Hcp1-induced apoptosis of HBMEC cells

**Figure 2:**
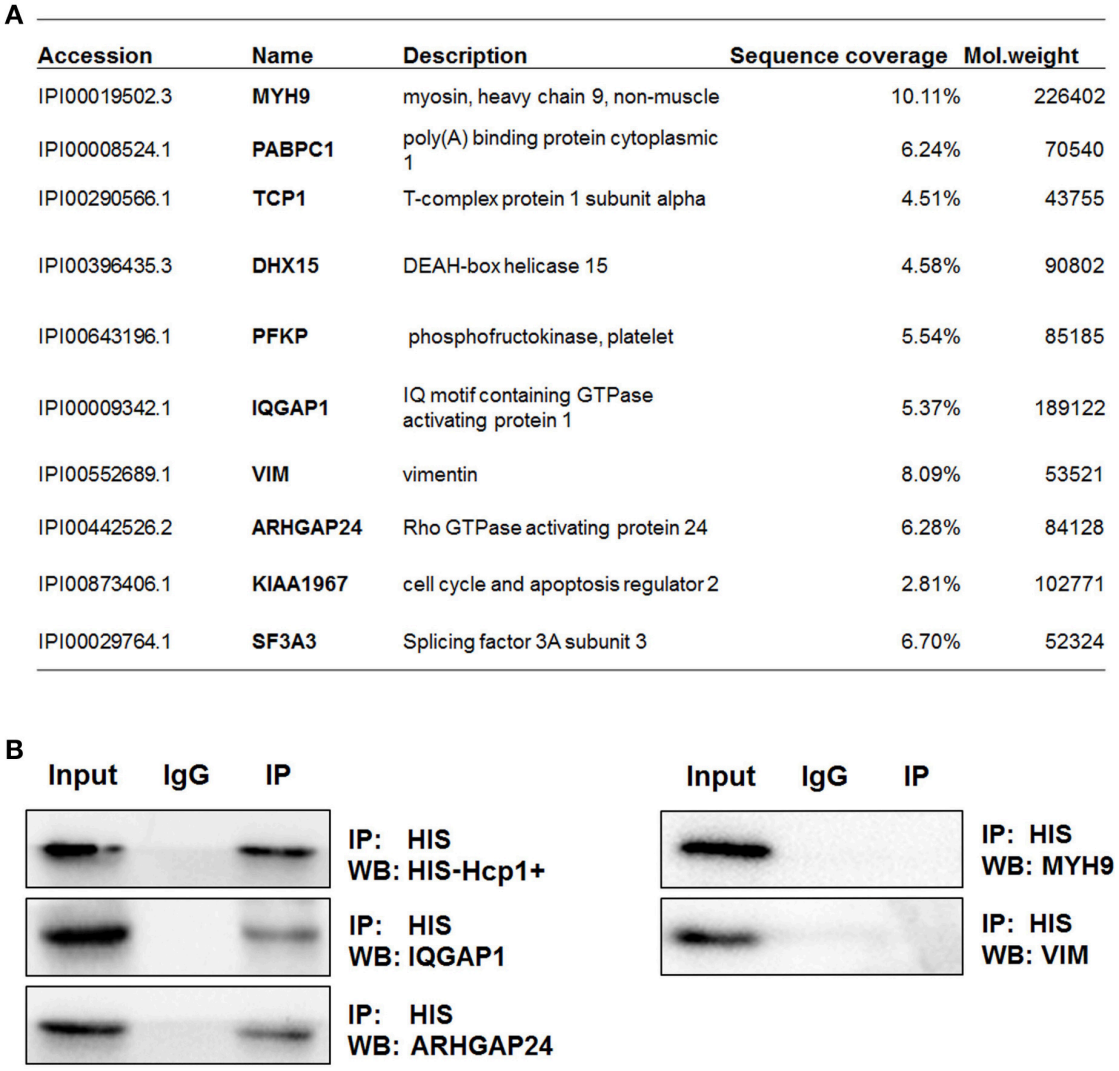
**IQGAP1 and ARHGAP24 can interact with Hcp1 in HBMEC cells. (A)** Putative binding partners of Hcp1 as identified by MS analysis. Proteins that could potentially bind to Hcp1 were co-immunoprecipitated by anti-His antibody, separated by SDS-PAGE and identified by MS. The matched epitope IgG was used as a negative control in the co-immunoprecipitation assay. **(B)** Detection of IQGAP1 (middle left) and ARHGAP24 (bottom left) in the Hcp1 immune complexes by anti-IQGAP1 and anti-ARHGAP24, respectively. IgG was used as a negative control. No binding of Hcp1 to either MYH9 (top right) or VIM (middle right) was observed.

**Figure 3:**
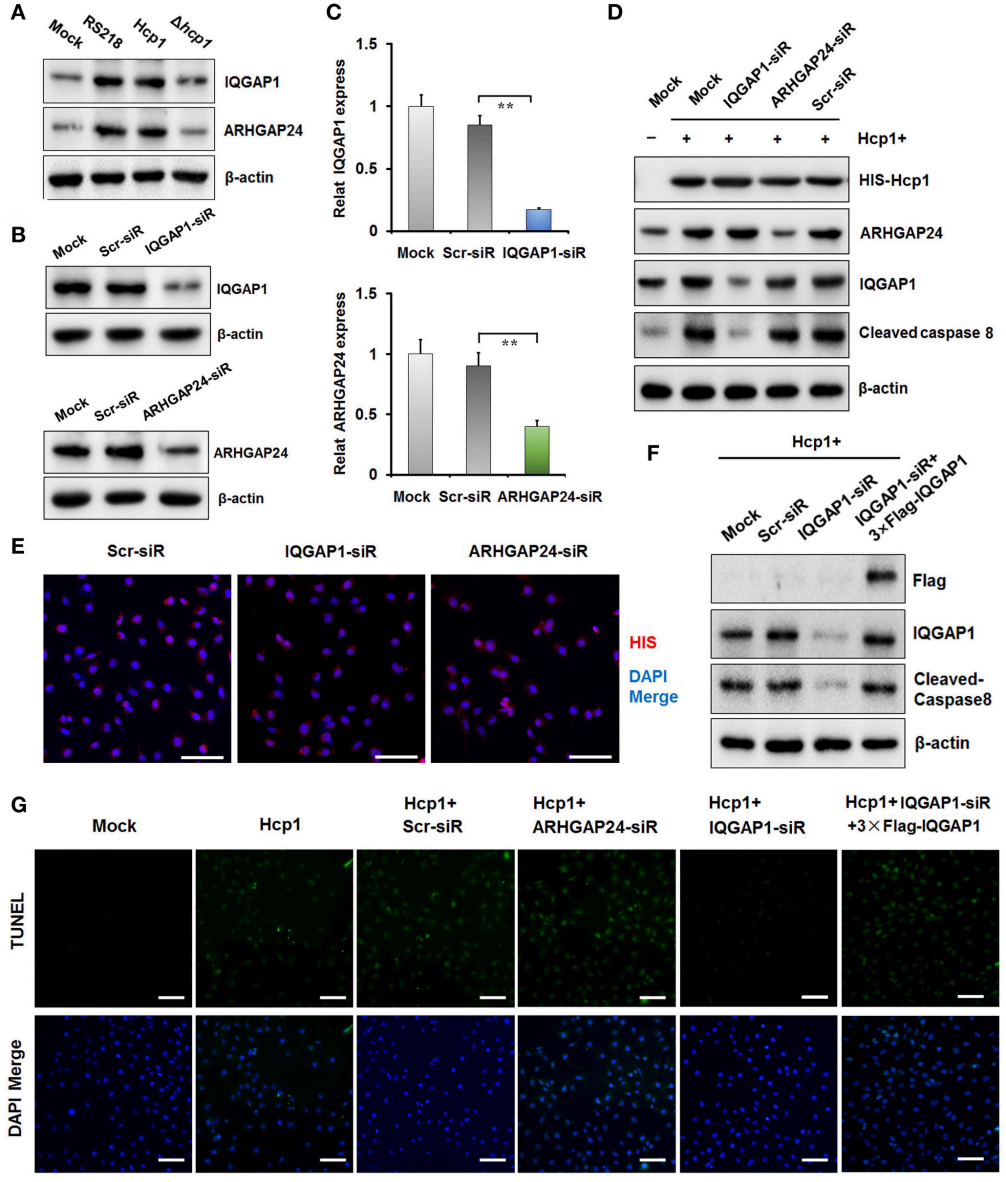
**IQGAP1 was involved in the modulation of HBMEC cell apoptosis by Hcp1. (A)** Up-regulation of both IQGAP1 (top) and ARHGAP24 (middle) in HBMEC cells incubated with wild-type RS218, RS218 mutant, or Hcp1 as demonstrated by Western blotting. β-actin was used as an internal standard. **(B,C)** Knockdown of IQGAP1 (left) and ARHGAP24 (right) by specific siRNAs. Quantitation of the relative mRNA and protein expression levels of IQGAP1 and ARHGAP24. **(D)** The expression levels of Hcp1 (panel 1), IQGAP1 (panel 2), ARHGAP24 (panel 3), and cleaved caspase 8 (panel 4) were measured by Western blotting (E) Immunofluorescence assay showing the uptake level of Hcp1 in HBMEC cells transfected with Scr-siR, IQGAP1-siRNA, or ARHGAP21-siRNA. Scale bar, 50 μm. **(F)** The expression levels of IQGAP1 (panel 2) and cleaved caspase 8 (panel 3) in Hcp1-treated HBMEC cells that were not transfected, transfected with Scr-siR, with IQGAP1-siRNA, or co-transfected with IQGAP1-siRNA and FLAG-tagged IQGAP1. **(G)** TUNEL assay showing the effect of IQGAP1 knockdown on reducing Hcp1-promoted apoptosis of HBMEC cells. Co-transfection of the IQGAP1-overexpression plasmid was shown to inhibitory effect of IQGAP1 knockdown on cell apoptosis. The average value ± s.d. of three separate experiments was plotted. ^**^P < 0.01. Scale bar, 50 μm.

To identify proteins that could potentially interact with Hcp1, we performed co-immunoprecipitation using anti-His antibody on His-tagged Hcp1-treated HBMEC cells. The isolated protein-antibody complexes were separated by SDS-PAGE and subjected individually to MS analysis. A panel of putative binding partners of Hcp1 with the highest percentages of sequence coverage was shown in Figure 2A. Among the listed protein targets, IQGAP1 and Rho GTPase activating protein 24 (ARHGAP24) are particularly noteworthy due to literature reports suggesting their implication in cell apoptosis (Liu Y. et al., 2013; Xu et al., 2016). Based on the above reasoning, we speculated that IQGAP1 and ARHGAP24 were likely involved in the mediation of HBMEC apoptosis initiated by Hcp1. The mixture of protein-Hcp1 complexes co-precipitated by anti-His antibody was detected via Western blot against a panel of antibodies targeting the putative candidates listed in Figure 2A. Both IQGAP1 and ARHGAP24 were confirmed by their respective antibodies to associate with Hcp1 (Figure 2B), whereas several other MS-detected candidates, including Myosin heavy chain 9 (MYH9) and Vimentin (VIM), were not observed and therefore considered as false positives (Figure 2B). We next evaluated the protein levels of IQGAP1 and ARHGAP24 in HBMEC cells in each experiment group. The results indicated that IQGAP1 and ARHGAP24 were similarly up-regulated in Hcp1-treated and RS218-infected cells (Figure 3A). However, neither candidate showed increased expression in HBMEC cells incubated with Hcp1-deleted RS218 over the uninfected control (Figure 3A). These results were consistent with the earlier observation of increased HBMEC cell apoptosis that followed the Hcp1 treatment (Zhou et al., 2012).

To further investigate the roles of IQGAP1 and ARHGAP24 in Hcp1-mediated apoptosis, we used small interference RNAs (siRNA) to separately generate IQGAP1- and ARHGAP24-knockdown HBMEC cell lines. As depicted in Figure 3E and Figure S1B, siRNA transfection had no appreciable effect on HBMEC cells' ability to uptake Hcp1. Meanwhile, the use of IQGAP1-siRNA resulted in significantly decreased protein and mRNA levels of IQGAP1 in HBMEC cells in comparison to the untreated control or those that were transfected with a mock siRNA (Figures 3B,C). Similarly, the protein and mRNA levels of ARHGAP24 in cells transfected with ARHGAP24-siRNA both declined in comparison to those in the control groups (Figures 3B,C). We then incubated the HBMEC cells with Hcp1 and measured the changes in the expression levels of cleaved caspase 3, 8, and 9, whose activation has been associated with blood-brain barrier damage (Tsai et al., 2015). While the expression of both cleaved caspase 3 and 9 showed no detectable change following the Hcp1 treatment, the level of caspase 8 was increased significantly in HBMEC cells incubated with Hcp1 compared to both the untreated control and those transfected with the scrambled siRNA (Figure 3D and Figure S1A). This was consistent with the TUNEL assay results, in which Hcp1 treatment was shown to increase the number of apoptotic HBMEC cells (Figure 3G). On the other hand, knockdown of IQGAP1 was found to negate the effect of Hcp1 on both the expression of cleaved caspase 8 (Figure 3D) and cell apoptosis (Figure 3G), whereas suppression of ARHGAP24 or transfection with the scrambled siRNA showed no observable impact on either. Importantly, the IQGAP1-knockdown phenotype could be rescued by co-transfection of a resistant IQGAP-overexpressing plasmid, in which case Hcp1 treatment again resulted in augmented expression of caspase 8 (Figure 3F) and increased apoptosis (Figure 3G). Taken together, we reasoned that IQGAP1 was likely to be a downstream effector of Hcp1 involved in modulating the HBMEC cell apoptosis.

### Hcp1 up-regulates IQGAP1 by rescuing it from ubiquitin-dependent degradation via substrate competition against smurf1

**Figure 4:**
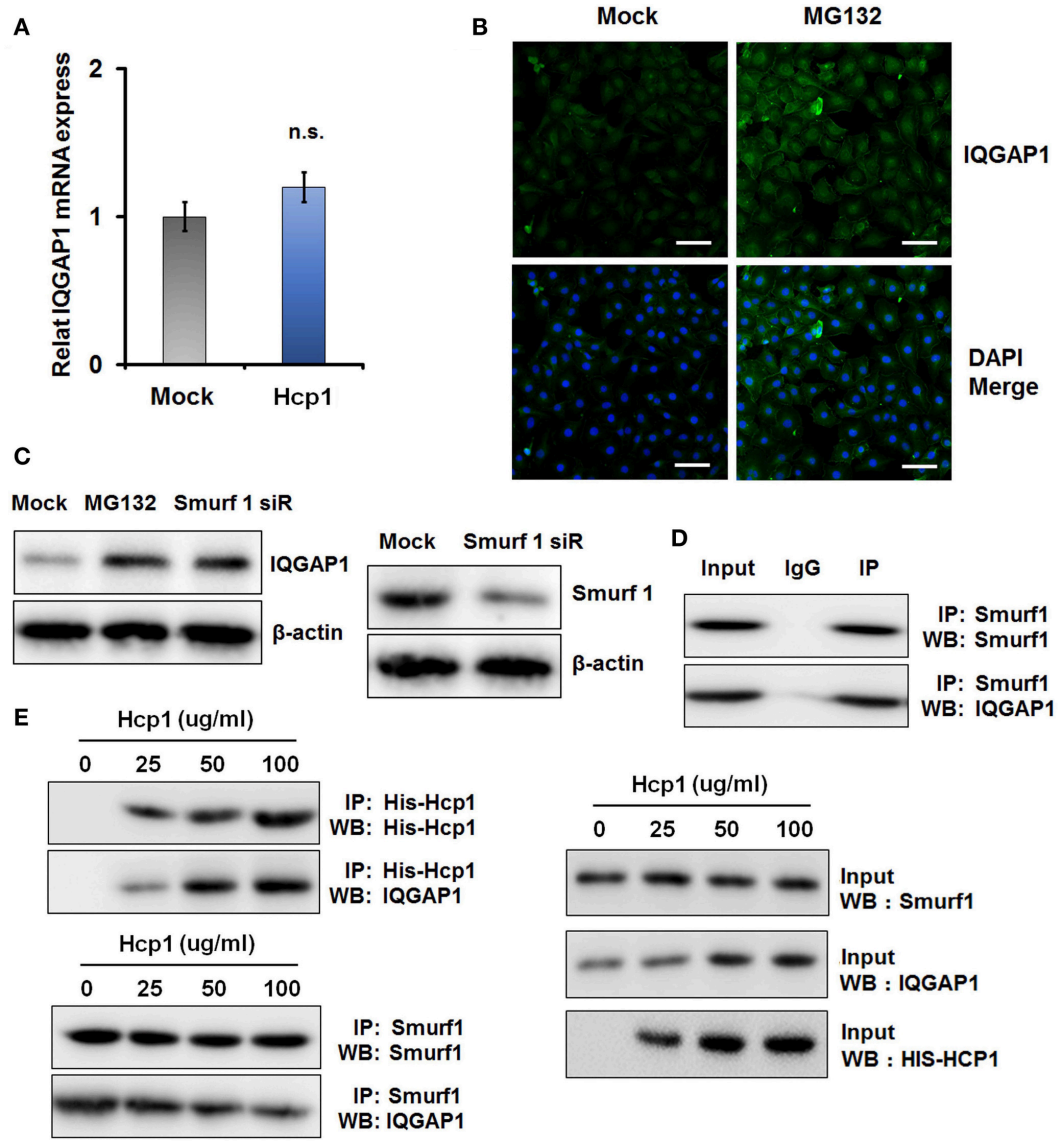
**Hcp1 can inhibit ubiquitin-dependent degradation of IQGAP1 by preventing it from binding to Smurf1 through substrate competition. (A)** mRNA expression levels of IQGAP1 in untreated and Hcp1-treated HBMEC cells. **(B)** HBMEC cells incubated with MG132, a proteasome-inhibiting agent, exhibited a higher cellular level of IQGAP1 compared to the untreated control. **(C)** Western blot showing that both MG132 and Smurf1 knockdown could significantly increase the protein level of IQGAP1 compared to the untreated control (left). The decrease of Smurf1 level as a result of siRNA transfection was verified by anti-Smurf1 antibody (right). **(D)** Co-immunoprecipitation experiment confirming the interaction between Smurf1 and IQGAP1. Proteins precipitated by anti-Smurf1 antibody were separated by SDS-PAGE and detected using anti-IQGAP1 antibody. IgG was used as an isotype control. **(E)** Hcp1 can compete against Smurf1 for binding to IQGAP1. HBMEC cells were incubated with increasing concentrations of Hcp1. Cell lysate samples (Input, right) were subjected to co-immunoprecipitation by anti-Smurf1 antibody, followed by Western blot detection using anti-IQGAP1 antibody (left). The fraction of Smurf1 binding IQGAP1 decreased in a dose-dependent manner with regard to the concentration of Hcp1 used. The increasing percentages of IQGAP1 binding to Hcp1 were verified by co-immunoprecipitation with anti-His antibody, followed by Western blot detection using anti-IQGAP1 antibody (left). Scale bar, 50 μm.

It is noteworthy that we did not observe any statistically significant difference between the mRNA level of IQGAP1 in Hcp1-treated HBMEC cells and that in the untreated control group (Figure 4A). This implied that IQGAP1 was regulated by Hcp1 not on the transcriptional, but on the translational or post-translational level. Based on the results of a previous study suggesting the co-localization of IQGAP1 and SMAD specific E3 ubiquitin protein ligase 1 (Smurf1) on plasma membrane (Liu C. et al., 2013), we speculated that Hcp1 could enhance the stability of IQGAP1 by preventing it from being targeted by ubiquitination. To test this possibility, we treated HBMEC cells with MG132, a cell-permeable proteasome inhibitor capable of suppressing the ubiquitin-dependent protein degradation pathway, and measured the intracellular concentration of IQGAP1. The fluorescence images indicated that incubation with MG132 caused IQGAP1 level to increase compared to the untreated control (Figure 4B). Furthermore, IQGAP1 was similarly upregulated in HBMEC cells transfected with Smurf1-siRNA (Figure 4C). Smurf1-IQGAP1 association was subsequently verified by co-immunoprecipitation using anti-Smurf1 antibody, followed by Western blot detection with anti-IQGAP1 antibody (Figure 4D). This binding was shown not to be the result of non-specific association with the IgG isotype. These results pointed to the likely scenario in which both Hcp1 and Smurf1 could bind to IQGAP1 in a competitive manner. Indeed, when we incubated HBMEC cells with increasing concentrations of His-Hcp1, we found that the fraction of IQGAP1 that was associated with Smurf1 declined in a dose-dependent manner as the percentage bound to Hcp1 increased (Figure 4E). The experimental data combined confirmed our speculation that Hcp1 could rescue IQGAP1 from being targeted by Smurf1 for ubiquitin-dependent degradation.

### IQGAP1 mediates Hcp1-dependent apoptosis of HBMEC cells by activating the MAPK signaling pathway

**Figure 5:**
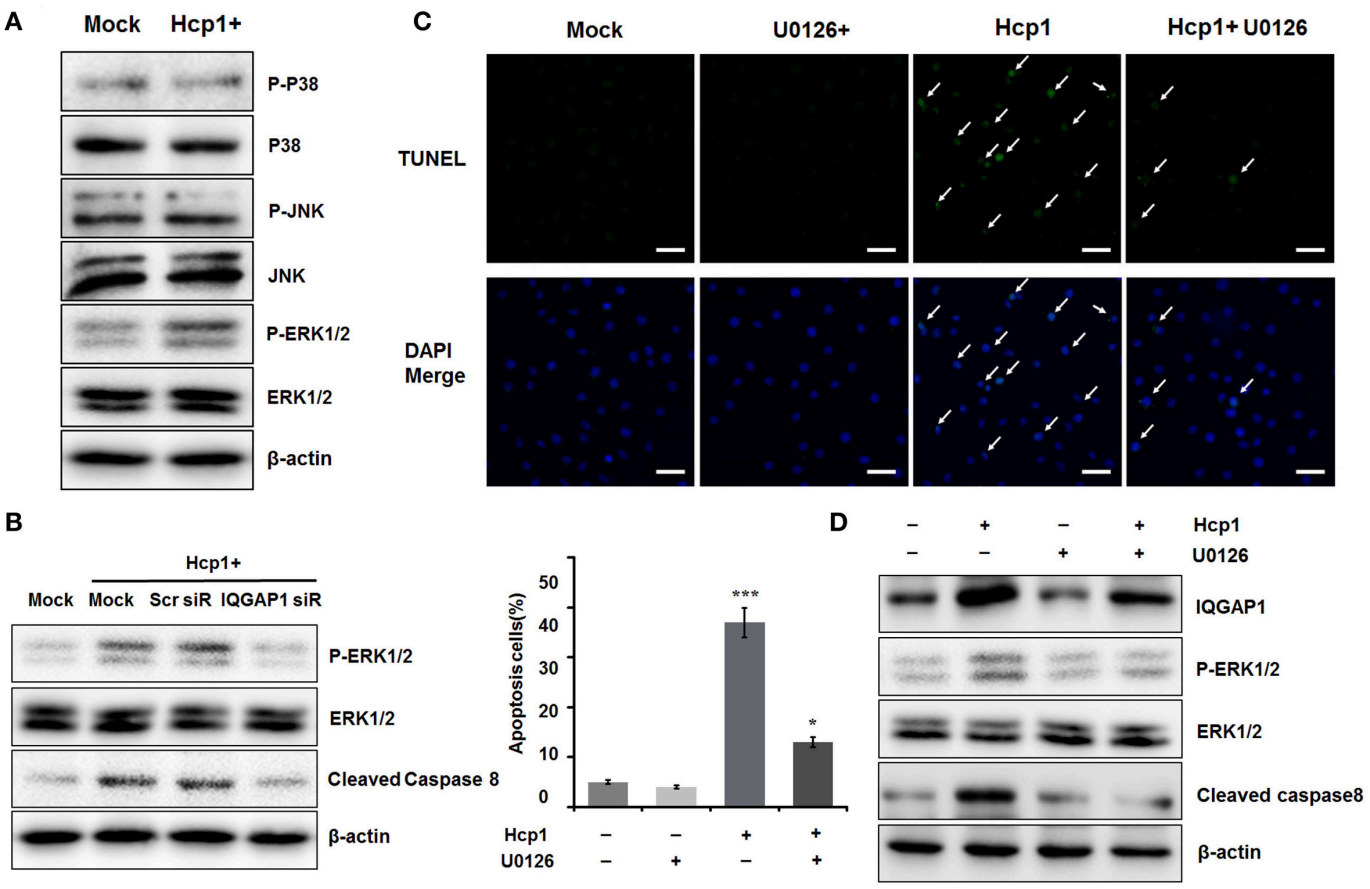
**IQGAP-promoted phosphorylation of ERK1/2 is responsible for Hcp1-induced apoptosis of HBMEC cells. (A)** Determination of the protein levels of total and phosphorylated P38 (panel 1–2), JNK (panel 3–4), and ERK1/2 (panel 5–6) in untreated (left) and Hcp1-treated (right) HBMEC cells. **(B)** IQGAP1 knockdown inhibited the Hcp1-induced up-regulation of cleaved caspase 8 and ERK1/2 phosphorylation compared to the Hcp1-treated control or Scr-siRNA-transfected cells. **(C)** TUNEL assay indicating that U0126, a MEK inhibitor, could significantly reduce cell apoptosis resulting from Hcp1 treatment (top). HBMEC cells treated with U0126 clone showed no significant apoptosis compared to the untreated control. The results are also shown in a column chart (*P* < 0.05) (bottom). **(D)** Western blot showing that both phosphorylated ERK1/2 and cleaved caspase 8 levels decreased in cells co-incubated with Hcp1 and U0126, compared to the ones treated with Hcp1 alone. The average value ± s.d. of three separate experiments was plotted. ^*^P < 0.05, ^***^P < 0.001. Scale bar, 50 μm.

Recently, it was reported that IQGAP1 could stimulate the MAPK signaling pathway and contribute to Angiotensin II (AngII)-induced apoptosis of podocytes (Liu Y. et al., 2013). To ascertain whether similar mechanism underlay the simulative effect of Hcp1 on HBMEC cell apoptosis, we evaluated the phosphorylation levels of several key members of the MAPK pathway, including P38, Jun N-terminal kinase (JNK), and ERK1/2, in Hcp1-treated HBMEC cells. Though we did not observe any significant alterations of P38 or JNK phosphorylation, Hcp1 treatment was found to cause a significant increase in the percentage of phosphorylated ERK1/2 (Figure 5A). Next, we examined the role of IQGAP1 in Hcp1-induced phosphorylation of ERK1/2. As seen in Figure 5B, while Hcp1 treatment was shown to increase both ERK1/2 phosphorylation and the expression of cleaved caspase 8, both effects were suppressed by siRNA-induced IQGAP1 knockdown in a specific manner. In comparison, transfection with a scrambled siRNA showed no detectable impact. We subsequently incubated HBMEC cells with U0126 (low concentration), an MAPK/ERK kinase (MEK) inhibitor (Ren and Guo, 2012; Deng et al., 2013). TUNEL assay indicated that incubation with U0126 alone did not lead to any detectable elevation of cell apoptosis compared to the control (Figure 5C). On the other hand, co-incubation with Hcp1 and U0126 reduced the number of apoptotic HBMEC cells by 67% compared to when only Hcp1 was included (Figure 5C). On a protein level, U0126 was also shown to be able to attenuate Hcp1-promoted up-regulation of ERK1/2 phosphorylation and of cleaved caspase 8 (Figure 5D). Noticeably, cells treated with Hcp1 alone expressed a roughly similar level of IQGAP1 as the ones co-incubated with both Hcp1 and U0126 (Figure 5D). This implied that U0126 was not involved in the direct regulation of either IQGAP1 or Hcp1. Taken together, we concluded that Hcp1-induced apoptosis of HBMEC cells was driven by IQGAP-mediated activation of the MAPK pathway.

### U0126, an MEK inhibitor, demonstrates protective effect against *E. coli* meningitis in a mouse model

**Figure 6:**
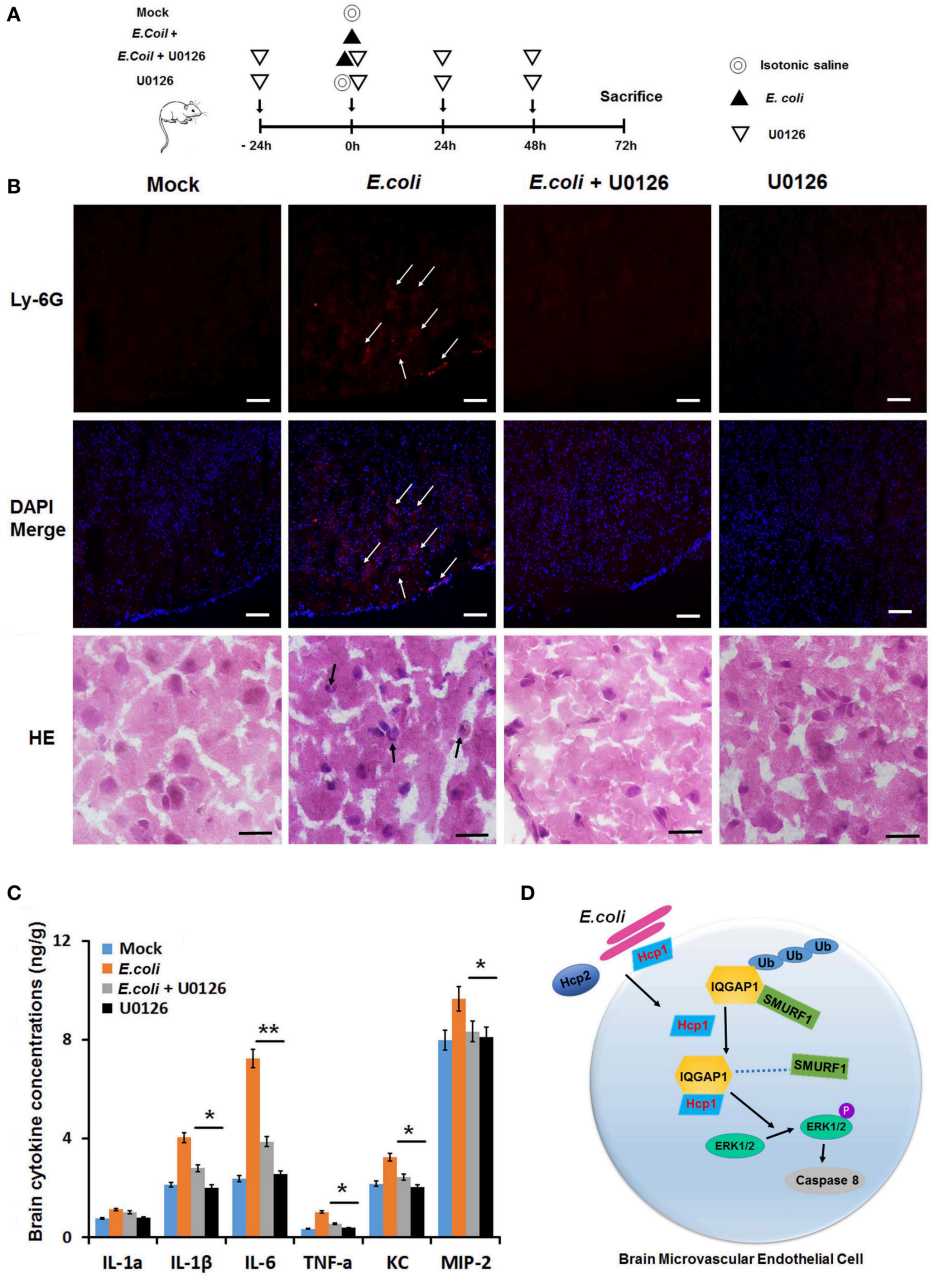
**U0126 can protect murine brain from *E. coli* meningitis. (A)** Schematic diagram illustrating the experimental design. **(B)** Inflammatory cell infiltration was evaluated by anti-Ly6G antibody(red) and H&E staining (black arrow). Injection of *E. coli* stimulated neutrophil infiltration in murine brain tissues (*E. coli* +); however, U0126 was shown to be able to effectively reduce *E. coli*-induced brain infection. IF scale bar, 100 μm, H&E staining scale bar, 10 μm. **(C)** Measurement of the concentrations of six inflammatory cytokines in murine brain tissues of four groups. **(D)** A mechanistic model illustrating the role of IQGAP1 in Hcp1-induced apoptosis of HBMEC cells. Hcp1 competes against Smurf1 for binding to IQGAP1, thereby rescuing the latter from ubiquitin-dependent degradation. On the other hand, IQGAP1 can promote the phosphorylation of ERK1/2, a key factor in the MAPK pathway responsible for cell apoptosis. The average value ± s.d. of three separate experiments was plotted. ^*^P < 0.05, ^**^P < 0.01.

The role of U0126 as a potential therapeutic agent against bacterial meningitis was then evaluated in a mouse meningitis model. C57BL/6 mice were randomly divided into four groups, the model group, the U0126 control group, the treatment group and the uninfected group. Mice in the model group and the treatment group were given an intranasal injection of *E. coli*, whereas those of the U0126 control group and the uninfected group received the same volume of isotonic saline. In addition, the animals in the U0126 control group and treatment group were intraperitoneally administered with 20 μM of U0126 (Figure 6A). Immunostaining with anti-Ly6G antibody demonstrated that while *E. coli* stimulated neutrophil infiltration in the murine brain tissues, the injection of U0126 led to a significant decrease in neutrophil recruitment indicative of alleviated cerebral infection (Figure 6B). We then measured the concentrations of several key inflammatory cytokines, including IL-1α, IL-1β, IL-6, TNF-α, KC, and MIP-2 in the abovementioned brain tissue samples. Consistent with the neutrophil staining results, all the tested brain cytokines were significantly up-regulated as a result of *E. coli* infection, but were down-regulated to baseline levels when U0126 was administered (Figure 6C). It should be stressed that treatment with U0126 alone produced no detectable effect on the expression of any of the abovementioned cytokines or on neutrophil infiltration (Figure 6C). Taken together, the experimental data confirmed the protective effect of U0126 against *E. coli* meningitis in the mouse model.

## Discussion

The current study aims to further elucidate the molecular mechanism that underlies *E. coli* meningitis. Building on our previous study suggesting the involvement of Hcp family proteins in the pathogenesis of *E. coli* meningitis, we provided direct experimental evidence showing that Hcp1, not Hcp2, was transported into the cytoplasm of HBMEC cells and responsible for triggering their apoptosis. With a combination of co-immunoprecipitation and MS detection, we then identified a panel of protein candidates that could potentially interact with Hcp1 and serve as its downstream factors. Further investigation revealed that IQGAP1 was mechanistically implicated in the stimulation of caspase 8 cleavage, thereby accelerating cell death. Hcp1 was found to increase the stability of IQGAP1 by preventing its association with Smurf1, thereby inhibiting its degradation through the ubiquitin cascade. Once up-regulated, IQGAP1 could promote the phosphorylation of ERK1/2 and thus activate the MAPK signaling pathway, leading to increased cell apoptotic activity.

IQGAP1 has been demonstrated to be able to elicit a diverse range of biological effects via binding to different protein partners (Mateer et al., 2003; Brown and Sacks, 2006). As a MAPK scaffolding protein, studies have shown that IQGAP1 can interact with several components in the MAPK signaling pathway, such as MEK1/2, ERK1/2, and B-Raf, which strongly suggests a role in cell apoptosis (Brown and Sacks, 2009). Indeed, IQGAP1 was reported to promote the phosphorylation of MEK1/2-ERK1/2 and AKT in a mouse model of chronic pressure overload (Sbroggio et al., 2011). Under these conditions, IQGAP1-knockout mice displayed significantly increased cardiomyocyte apoptosis. In another study, suppression of IQGAP1 expression in podocytes via siRNA knockdown led to a marked decline in AngII-induced phosphorylation of ERK1/2 and concomitantly decreased cell apoptosis (Liu Y. et al., 2013). Proteomic analysis based on immunoprecipitation coupled with MS detection revealed that IQGAP1 could bind to mammalian intracellular ribonuclease L (RNase L), which might modulate JNK phosphorylation, and ultimately, cell apoptosis (Sato et al., 2010). Studies have also revealed that IQGAP1 could stimulate *E. coli* invasion of HBMEC cells by interacting with β-catenin and promoting its dissociation from adherens junction (Krishnan et al., 2012). In our study, the ability of IQGAP1 to mediate Hcp1-promoted HBMEC cell apoptosis can also be traced back to its involvement in the MAPK pathway. On the other hand, our results suggested that IQGAP1 was likely to be sequestered by Smurf1 for ubiquitination and proteasomal degradation under normal physiological conditions; however, Hcp1 could stabilize IQGAP1 by competitively inhibiting its interaction with Smurf1. In consistent with our current results, IQGAP1 was shown to target TGF-β receptor II (TβRII) in hepatic stellate cells leading to its recognition by Smurf1 (Liu Y. et al., 2013). This finding led to the subsequent observation that Hcp1 could compete against Smurf1 for the binding of IQGAP1. Therefore, it appeared that the role of Hcp1 to up-regulate IQGAP1 was attributable to its ability to prevent the latter from being targeted by Smurf1 toward the ubiquitination cascade.

Although the main treatment strategy against bacterial meningitis centers on the eradication of the causative agent via the use of antibiotics, the finding that persistent inflammation can cause further and often irreversible damage to the impacted brain tissues has prompted efforts to develop adjunctive therapeutic strategies. In one study, high mobility group box 1 was suggested as such a target due to its role in the development of pneumococcal meningitis and its ability to elicit strong inflammatory response in the injured brain cells (Hohne et al., 2013). In a mouse model, immunization with α-glycerophosphate oxidase showed benefit against invasive *pneumococcal* meningitis (Mahdi et al., 2012). Recently, the MAPK signaling pathway was seen as a potential therapeutic target in meningitis treatment. It was found that *Group B Streptococcus* infection could stimulate the MAPK-dependent expression of the transcriptional repressor Snail1, leading to increased blood-brain barrier permeability and facilitated penetration of infectious bacteria (Kim et al., 2015). The pathological significance of the MAPK signaling pathway was also demonstrated in this study through the finding that its activation by IQGAP1 was a crucial step in the development of *E. coli* meningitis. Based on these results, we decided to test the efficacy of U0126 against Hcp1/IQGAP1-promoted apoptosis of HBMEC cells. U0126 is a well-established inhibitor of MEK1 and MEK2 in the MAPK signaling pathway(Brindle, 2012) and has been applied as a therapeutic agent in various studies with demonstrated effect. Treatment with U0126 was found to suppress the growth of two embryonal rhabdomyosarcoma cell lines (Marampon et al., 2009). The clinical efficacy of U0126 against renal tumors was also evidenced by imaging studies in an endogenous mouse model (Flores et al., 2013). The fact that U0126 could abolish the stimulating effect of IQGAP1 upregulation on the inflammatory response of the infected brain tissues lent further experimental credence to the involvement of the MAPK pathway in Hcp1-promoted *E. coli* meningitis. Meanwhile, this finding also highlighted the clinical potential of U0126 for adjunctive therapy against the disease.

In summary, we have identified IQGAP1 as a key mediator implicated in the pathogenesis of *E. coli*-induced meningitis. In the event of *E. coli* infection, cellular IQGAP1 can be up-regulated by the inoculation of Hcp1, which in turn leads to enhanced MAPK pathway activity and increased cell apoptosis. We have also demonstrated that inhibiting the MAPK pathway could provide a potentially valuable therapeutic solution to the treatment of bacterial meningitis.

## Author contributions

JL and YZ conceived and designed the experiments. MZ, LZ, YZ, SL, CZ, LW, and HC performed the experiments. MZ, LZ, and YZ wrote and revised the paper. MZ, LZ, YZ, and SL analyzed the data. JL and YZ supervised the project. All authors reviewed the paper.

## Funding

This work was supported by the National Natural Science Foundation of China (81301393, 81672833).

### Conflict of interest statement

The authors declare that the research was conducted in the absence of any commercial or financial relationships that could be construed as a potential conflict of interest.
