# Supplementary material for: IQGAP1 Mediates Hcp1-Promoted Escherichia coli Meningitis by Stimulating the MAPK Pathway
Source: Front Cell Infect Microbiol. 2017 Apr 19;7:132. doi: 10.3389/fcimb.2017.00132 (PMC5395654; doi:10.3389/fcimb.2017.00132)
Supplement: Supplementary file 1 [file DataSheet1.DOCX]

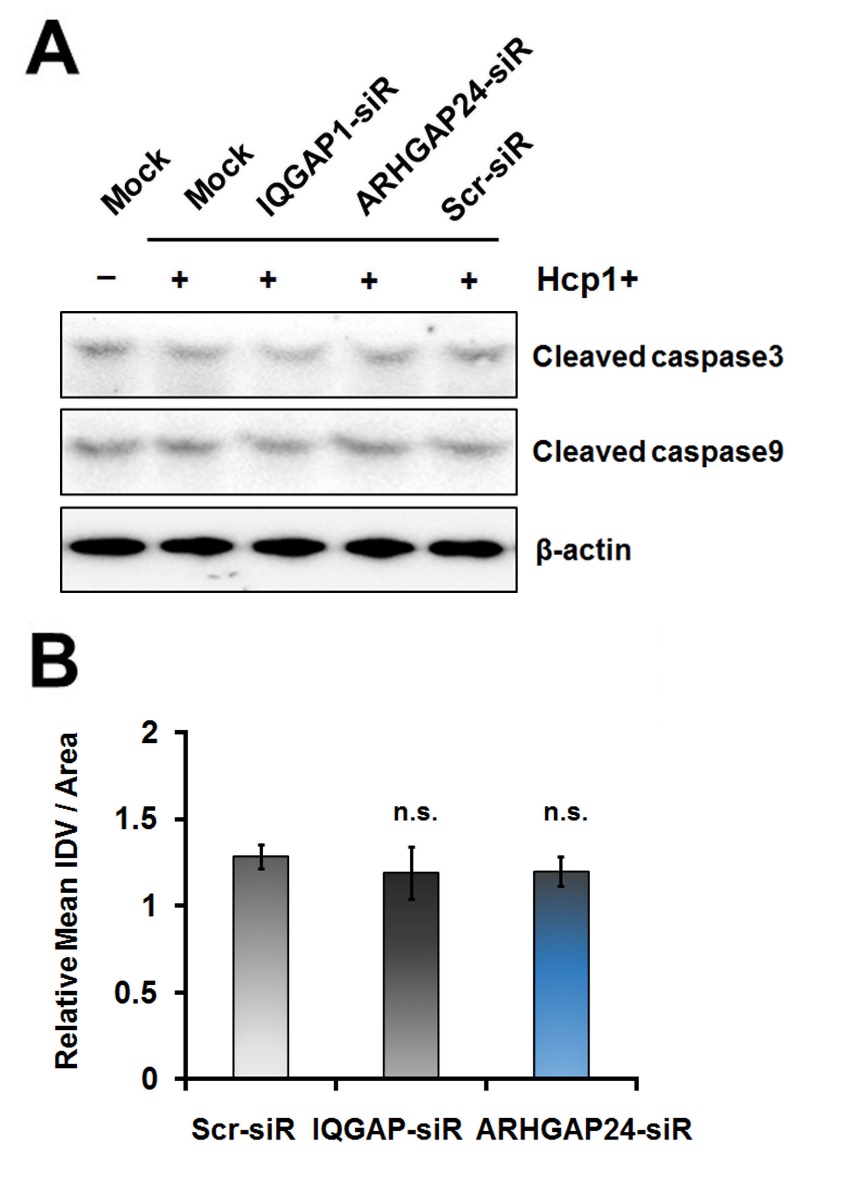


Figure S1: (A) The expression levels of cleaved caspase 3 and cleaved caspase 9 were measured by Western blotting. β-actin was used as an internal standard. (B) Relative uptake levels of Hcp1 (red channel) in figure 3E were quantified and converted to mean IDV.
